# The length of ribosomal binding site spacer sequence controls the production yield for intracellular and secreted proteins by *Bacillus subtilis*

**DOI:** 10.1186/s12934-020-01404-2

**Published:** 2020-07-29

**Authors:** Kristina Volkenborn, Laura Kuschmierz, Nuka Benz, Patrick Lenz, Andreas Knapp, Karl-Erich Jaeger

**Affiliations:** 1 Institute of Molecular Enzyme Technology, Heinrich Heine University Düsseldorf, Forschungszentrum Jülich GmbH, 52425 Jülich, Germany; 2 Present Address: Environmental Microbiology and Biotechnology-Molecular Enzyme Technology and Biochemistry, University Duisburg-Essen, 45141 Essen, Germany; 3 Institute of Bio- and Geosciences IBG-1: Biotechnology, Forschungszentrum Jülich GmbH, 52425 Jülich, Germany; 4 Bioeconomy Science Center (BioSC), C/o Forschungszentrum Jülich, 52425 Jülich, Germany

**Keywords:** *Bacillus subtilis*, Production optimization, Translation initiation, Ribosome binding site, Spacer

## Abstract

**Background:**

*Bacillus subtilis* is widely used for the industrial production of recombinant proteins, mainly due to its high secretion capacity, but higher production yields can be achieved only if bottlenecks are removed. To this end, a crucial process is translation initiation which takes place at the ribosome binding site enclosing the Shine Dalgarno sequence, the start codon of the target gene and a short spacer sequence in between. Here, we have studied the effects of varying spacer sequence lengths in vivo on the production yield of different intra- and extracellular proteins.

**Results:**

The shuttle vector pBSMul1 containing the strong constitutive promoter P_*HpaII*_ and the optimal Shine Dalgarno sequence TAAGGAGG was used as a template to construct a series of vectors with spacer lengths varying from 4 to 12 adenosines. For the intracellular proteins GFPmut3 and β-glucuronidase, an increase of spacer lengths from 4 to 7–9 nucleotides resulted in a gradual increase of product yields up to 27-fold reaching a plateau for even longer spacers. The production of secreted proteins was tested with cutinase Cut and swollenin EXLX1 which were N-terminally fused to one of the Sec-dependent signal peptides SPPel, SPEpr or SPBsn. Again, longer spacer sequences resulted in up to tenfold increased yields of extracellular proteins. Fusions with signal peptides SPPel or SPBsn revealed the highest production yields with spacers of 7–10nt length. Remarkably, fusions with SPEpr resulted in a twofold lower production yield with 6 or 7nt spacers reaching a maximum with 10–12nt spacers. This pattern was observed for both secreted proteins fused to SPEpr indicating a dominant role also of the nucleotide sequence encoding the respective signal peptide for translation initiation. This conclusion was corroborated by RT qPCR revealing only slightly different amounts of transcript. Also, the effect of a putative alternative translation initiation site could be ruled out.

**Conclusion:**

Our results confirm the importance of the 5′ end sequence of a target gene for translation initiation. Optimizing production yields thus may require screenings for optimal spacer sequence lengths. In case of secreted proteins, the 5′ sequence encoding the signal peptide for Sec-depended secretion should also be considered.

## Background

*Bacillus subtilis* is one of the most important Gram-positive bacteria for the industrial production of recombinant proteins and enzymes [1]. The type strain *B. subtilis* 168 belongs to the best studied bacteria allowing to devise strategies for production optimization at many different stages of transcription, protein biosynthesis and maturation [2]. For example, more than 114 endogenous putative promoters found in *B. subtilis* were assayed for their transcription strengths in different growth phases [3]. Furthermore, libraries of N-terminal Sec secretion-dependent signal peptides (SP) can easily be screened to identify the best out of more than 170 different SPs for secretion of a target protein [4–7]. Translation and, in particular, its initiation represents another limiting factor for recombinant protein production [8] which was targeted so far in only few studies with *B. subtilis*.

Translation initiation takes place at the ribosome binding site (RBS) which is located within the 5′ untranslated region (5′UTR) of an mRNA (reviewed for example in [8, 9]). The RBS encloses the Shine Dalgarno (SD) sequence, the start codon of the target gene and a short spacer sequence in between. Base pairing of the SD sequence with the anti-SD sequence located at the 3′ terminus of the 16S rRNA in the 30S small ribosomal subunit initiates the formation of the translation machinery [10, 11]. Within this initiation complex, the start codon is directed into the ribosomal P-site. Formation of this complex and thus protein production can be improved by so called “strong” SD sequences with high affinity to the respective anti-SD sequence and use of the most frequently occurring start codon AUG [12]. The spacer sequence between SD sequence and start codon is assumed to bridge the spatial distance between SD sequence and P-site and its optimal length of 7–9nt was determined for the intracellular production of LacZ in *B. subtilis* [12]. Furthermore, nucleotides flanking the RBS up- and downstream can form mRNA secondary structures that mask the RBS and thereby impede translation initiation [13]. Also, the 5′ sequence of the target gene can influence these secondary structures, and studies of translation initiation in *E. coli* indicate that rare codons in the 5′ sequence of the target gene are not important for deceleration of translation elongation but rather for reducing secondary structures concealing the RBS [14]. Consequently, RBS sequences which were identified as optimal for the production of one target protein cannot be transferred necessarily to another target protein as demonstrated in a study that optimized the production of an intracellular laccase and an extracellular protease [15]. Calculating RNA secondary structures in silico with tools like RNAfold [16] or RBS calculator [17, 18] can help to optimize the production yield of recombinant proteins but they address just one out of several important steps of the complex protein biosynthesis pathway. Here, we present a systematic study to resolve in vivo the effect of the spacer sequence lengths on the production yields of different intra- and extracellular proteins by *B. subtilis*. Furthermore, we have analyzed whether optimal spacer sequence lengths can be predicted and transferred to optimizing the production yield of other proteins.

The study was performed using *B. subtilis* TEB1030 which is based on the strain 168 derivative DB430 lacking four extracellular (AprE, Bpr, Epr, NprE) and one intracellular protease (IspA) to avoid proteolytic degradation of the target proteins [19, 20] and additionally lacking both extracellular lipases LipA and LipB [21]. Genes cloned into the expression plasmid pBSMul1 [22] allowing for high gene copy numbers and strong, constitutive expression [23] under control of promoter P_*HpaII*_. The constructed vector series carries spacers of 4–12nt lengths and we quantified the production of two intracellular and two secreted proteins by SDS-PAGE, activity and split GFP assays. As intracellular proteins, GFPmut3 [24] and the β-glucuronidase UidA from *E. coli* (here termed GUS, [25]) and as secreted proteins, *Fusarium solani pisi* cutinase Cut and *B. subtilis* swollenin EXLX1 fused to the signal peptides SPPel, SPBsn or SPEpr were produced. Finally, the experimental results from this systematic study were compared to in silico calculated translation initiation rates. The data presented here can serve to optimize protein production using optimal spacer sequence lengths.

## Methods

### Media and culturing conditions

*E. coli* DH5α [26] and *B. subtilis* TEB1030 [21] were grown at 37 °C in shaking flasks with 1/10 volume of LB medium (10 g/l tryptone, 10 g/l NaCl, 5 g/l yeast extract) containing either 100 µg/ml ampicillin (*E. coli*) or 50 µg/ml kanamycin (*B. subtilis*).

### Transformation of *E. coli* and *B. subtilis*

Transformation of chemical competent *E. coli* DH5α and of *B. subtilis* TEB1030 protoplasts was carried out as described previously [27, 28].

### Expression cultures

For the expression of target genes, a 10 ml overnight culture was inoculated with a single *B. subtilis* transformant and grown at 37 °C under aerobic conditions. This pre-culture was used to inoculate a 10 ml main-culture to a cell density (OD_580nm_) of 0.05. The expression cultures were grown at 37 °C for 6 h under aerobic conditions. Subsequently, cell density was measured and cells were separated from the supernatant by centrifugation (21,000*×g*, 10 min) if necessary for further analyses.

### Molecular cloning

Cloning of genes was performed using standard molecular methods [27]. Kits for the purification of nucleic acids were purchased from Analytic Jena (Jena, Germany) and enzymes were purchased from Thermo Fisher Scientific (St. Leon-Roth, Germany).

### Construction of standard expression plasmids

Target genes for the construction of the standard expression plasmids (Table 1) were amplified as *Nde*I/*Xba*I fragments with primers listed in Additional file 1: Table S1 from different templates: *GFPmut3* was taken from a modified pEBP41 [29] where we deleted an intrinsic *Nde*I site by QuikChange PCR^®^ [30] using the primers P1 and P2. The *GUS* gene (*uidA*) was amplified from *E. coli* DH5α genomic DNA. Fusions of *EXLX1*-*11* and *cut*-*11*, respectively, with the signal peptide sequences *SPepr*, *SPpel* and *SPbsn* were amplified from a previously constructed signal peptide library [31]. All gene fragments were ligated into the *Nde*I/*Xba*I hydrolyzed *E.* *coli*–*B.* *subtilis* shuttle vector pBSMul1 [22]. This standard expression plasmid contains a 4 nucleotide spacer and is therefore termed pBS4nt in this study.

**Table 1:** Plasmids used in this study

Name	Description	Source
pBSMul1	*E.* *coli*–*B.* *subtilis* shuttle vector, P_*HpaII*_, secretion signal (*SPlipA*), ColE1 *repB Km*^*r*^*Amp*^*r*^	[22]
pET22-*sfGFP1*-*10*	pET22b(+) containing the truncated *sfGFP1*-*10* gen under control of P_*T7*_	[31]
pEBP41	*E.* *coli*–*B.* *subtilis*–*P.* *putida* shuttle vector, P_*T7*_, P_*xyl*_, *Gm*^*r*^, *GFPmut3*	[29]
pEBP41-*Nde*I	pEBP41 with deleted *Nde*I site within the *GFPmut3 gene* by QuikChange PCR^®^ using the primers P1 and P2	This study
pBSMul1(SPBox)-*SPxxx*-*cut*-*11*	Signal peptide (SP) library based on pBSMul1 containing *Hin*dIII–*Xba*I inserts of the cutinase *cut* of *F. solani pisi* fused to a *GFP11* tag and with different signal peptides (*SPepr*, *SPpel*, *SPbsn*)	[31]
pBSMul1(SPBox)-*SPxxx*-*EXLX1*-*11*	Signal peptide (SP) library based on pBSMul1 containing *Hin*dIII–*Xba*I inserts of the swollenin *EXLX1* of *B. subtilis* fused to a *GFP11* tag and with different signal peptides (*SPepr*, *SPpel*, *SPbsn*)	[31]
Standard expression plasmids (based on pBSMul1 with a 4 nucleotide spacer)		
pBS4nt-*GFPmut3*	pBSMul1 containing a 723 bp *Nde*I–*Xba*I *GFPmut3* fragment amplified from pEBP41-*Nde*I with primers P3/P4	This study
pBS4nt-*GUS*	pBSMul1 containing a 1828 bp *Nde*I–*Xba*I (*uidA*) fragment amplified from *E. coli* DH5α genome with primers P5/P6	This study
pBS4nt-*SPepr*-*cut*-*11*	pBSMul1 containing an 845 bp *Nde*I–*Xba*I *SPepr*-*cut*-*11* fragment amplified from SP library using primers P7/P10	This study
pBS4nt-*SPpel*-*cut*-*11*	pBSMul1 containing an 827 bp *Nde*I–*Xba*I *SPpel*-*cut*-*11* fragment amplified from SP library using primers P8/P10	This study
pBS4nt-*SPbsn*-*cut*-*11*	pBSMul1 containing an 848 bp *Nde*I–*Xba*I *SPbsn*-*cut*-*11* fragment amplified from SP library using primers P9/P10	This study
pBS4nt-*SPepr*-*EXLX1*-*11*	pBSMul1 containing an 824 bp *Nde*I–*Xba*I *SPEXLX1*-*cut*-*11* fragment amplified from SP library using primers P7/P10	This study
pBS4nt-*SPpel*-*EXLX1*-*11*	pBSMul1 containing an 806 bp *Nde*I–*Xba*I *SPpel*-*EXLX1*-*11* fragment amplified from SP library using primers P8/P10	This study
pBS4nt-*SPbsn*-*EXLX1*-*11*	pBSMul1 containing an 827 bp *Nde*I–*Xba*I *SPbsn*-*EXLX1*-*11* fragment amplified from SP library using primers P9/P10	This study
Expression plasmids with extended spacers		
pBSxnt-*Y*	Spacers ranging in length from 5 to 12 nucleotides (xnt) by insertion of adenosines for all above mentioned plasmids (Y: -*GFPmut3,* -*GUS,* -*SPepr*-*cut*-*11,* -*SPpel*-*cut*-*11,* -*SPbsn*-*cut*-*11,* -*SPepr*-*EXLX1*-*11,* -*SPpel*-*EXLX1*-*11,* -*SPbsn*-*EXLX1*-*11*)	This study
pBSxnt-*SPepr*-*cut*-*11*_start1	pBSxnt-*SPepr*-*cut*-*11* with the second putative translational start codon ATG exchanged by ACG	This study
pBSxnt-*SPepr*-*cut*-*11*_start2	pBSxnt-*SPepr*-*cut*-*11* with the first translational start codon ATG exchanged by ACG	This study
Spacer sequence library		
pBS7nt-4N-*GFPmut3*	Spacer library based on pBS7nt-*GFPmut3* containing the 7nt long spacer sequence 5′-NNNNCAT-3′ inserted by QuikChange PCR^®^ and primers P27/P28	This study
pBS7nt-4N-*SPpel*-*cut*-*11*	Spacer library based on pBS7nt-*SPpel*-*cut*-*11* containing the 7nt long spacer sequence 5′-NNNNCAT-3′ inserted by QuikChange PCR^®^ and primers P29/P30	This study

### Construction of expression vectors with different spacer sequence lengths

The spacer sequence between the SD sequence and the start codon was extended by insertion of adenosines in the spacer sequence of the pBS4nt-*SPepr*-*cut*-*11* vector using QuikChange PCR^®^ [30] and the primers P11–P26 (Additional file 1: Table S1). The resulting vector series contains spacer sequences with lengths between 5 and 12nt (as indicated by xnt in the plasmid name). Subsequently, the vector series pBSxnt-*SPepr*-*cut*-*11* was hydrolyzed using the restriction enzymes *Nde*I and *Xba*I, and *Nde*I/*Xba*I fragments of target genes *GFPmut3, GUS, SPpel*-*cut*-*11, SPbsn*-*cut*-*11, SPepr*-*EXLX1*-*11, SPpel*-*EXLX1*-*11* and *SPbsn*-*EXLX1*-*11* were ligated into these plasmids to construct a vector series with different spacer lengths for each target gene.

### Mutagenesis of spacer sequences and spacer library screening

**Fig. 1:**
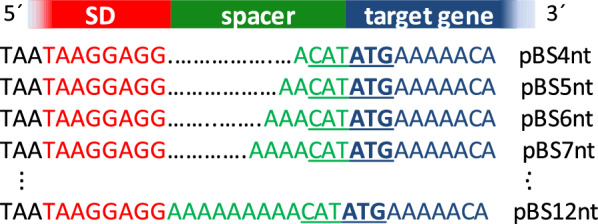
Nucleotide composition of ribosome binding sites of plasmids pBS4nt to pBS12nt depicting different spacer lengths. Shine Dalgarno (SD) sequence (red), spacer (green) and start of target gene (blue) are highlighted. The cleavage site for *Nde*I including the start codon (bold) is underlined. Spacer sequences were varied in length from 4 to 12nt by insertion of adenosines. The original plasmid pBSMul1 is designated here as pBS4nt to indicate the length of the spacer

The spacer sequence AAAACAT of pBS7nt-*GFPmut3* and pBS7nt-*SPpel*-*cut*-*11* was replaced by NNNNCAT (N = A, T, C, G) preserving CAT and thus the *Nde*I restriction site of the expression plasmid (see Fig. 1) using QuikChange PCR^®^ [30] and the primer pairs P27/P28 and P29/P30, respectively (Additional file 1: Table S1). After transformation of *E.* *coli* DH5α, about 2000 single clones for each target gene were washed off from agar plates and the plasmid DNA was isolated resulting in the libraries pBS7nt-4N-*GFPmut3* and pBS7nt-4N-*SPpel*-*cut*-*11*. Libraries were introduced in *B. subtilis* TEB1030 and 908 clones producing GFPmut3 variants as well as 828 clones producing SPPel-Cut-11 variants were cultivated as pre-culture in microtiter plate scale (200 µl selective medium, 37 °C, 900 rpm, 16 h). Main cultures were prepared as 20-fold dilution of the pre-cultures with fresh selective medium, cultivated for 6 h under the same conditions and subsequently assayed for intracellular GFP fluorescence or extracellular Cut-11 (lipolytic activity and split GFP assay). The best performing clones were cultivated as biological triplicates in shake flasks (see section “Expression cultures” above) with the standard 7nt constructs as reference.

### Construction of plasmids with different translation start sites

To exchange one of the two different translational start codons (ATG) by ACG in the plasmid series pBSxnt-*SPepr*-*cut*-*11*, a QuikChange mutagenesis [30] was performed with primer pairs P29/30, P31/32, P33/34, P35/36, P37/38, P39/40, P41/42, P43/44, P45/46 or P47/48 to exchange the first ATG in each of spacer variants (resulting in pBSxnt-*SPepr*-*cut*-*11*_start2), or with primer pair P49/50 to exchange the second ATG in all spacer variants (resulting in pBSxnt-*SPepr*-*cut*-*11*_start1).

### Split GFP assay

The amount of secreted cutinase Cut-11 and swollenin EXLX1-11 was detected by the split GFP assay. The truncated GFP1-10 fragment (detector) was produced by *E.* *coli* BL21(DE3) with pET22-*sfGFP1*-*10* as described previously [31]. 20 µl culture supernatant was mixed with 180 µl detector solution and incubated at room temperature for at least 16 h. Fluorescence was measured using the Tecan Infinite M1000 Pro microplate reader (Tecan, Männedorf, Switzerland). The following parameters were used for fluorescence measurements: λ_Ex_ = 485 nm (bandwidth 10 nm), λ_Em_ = 505–550 nm (5 nm steps, bandwidth 5 nm, gain 120). The emission maximum at 510 nm was used for calculation of relative fluorescence units.

### Cutinase activity assay

The lipolytic activity of Cut-11 was measured using the chromogenic substrate *p*-nitrophenyl-palmitate (*p*NPP) as described by Winkler and Stuckmann [32]. To prepare the substrate solution, 15 mg *p*NPP (Sigma-Aldrich/Merck, Darmstadt, Germany) were dissolved in 5 ml isopropanol and mixed with 45 ml Sørensen buffer pH 8 (47.22 mM Na_2_HPO_4_, 2.77 mM KH_2_HPO_4_, 1.11 mg/ml gum arabic, 2.3 mg/ml sodium deoxycholic acid). Culture supernatants were diluted tenfold with 50 mM Tris-HCl (pH 8). 10 µl of the diluted culture supernatants were mixed with 190 µl substrate solution, incubated at 37 °C and the change of absorption at 410 nm was measured for 15 min using the SpectraMax 250 plate reader (Molecular Devices, Biberach an der Riss, Germany). Volumetric activities (U/ml) were calculated with the molar absorption coefficient of *p*NP (15,000 M^−1^ cm^−1^ for the used reaction parameters) and subsequently normalized to the cell density (OD_580_).

### GUS activity assay

Enzymatic activity of GUS was determined with the chromogenic substrate *p*-nitrophenyl-glucuronide (*p*NPG, Sigma-Aldrich/Merck, Darmstadt, Germany) as described by Cui et al. [33]. For subsequent cell lysis, 30 µl of the GUS expression cultures were mixed with 85 µl PBS buffer (137 mM NaCl, 2.7 mM KCl, 8 mM Na_2_HPO_4_ 2H_2_O, 1.76 mM KH_2_PO_4_, pH 7.4) and 5 µl of a 1 mg/ml lysozyme solution in PBS and incubated at 37 °C for 30 min. The cell lysate was diluted 20-fold with PBS buffer and 50 µl of the dilution were mixed with 50 µl of substrate solution (1.59 mM *p*NPG in PBS) and incubated at 37 °C for 2 min. The reaction was stopped by addition of 100 µl 1 M Na_2_CO_3_. The adsorption at 410 nm was measured using the SpectraMax 250 plate reader (Molecular Devices, Biberach an der Riss, Germany). The volumetric activity (U/ml) was calculated using the molar absorption coefficient of *p*NP (15,301 M^−1^ cm^−1^ for the used reaction conditions) and normalized to the cell density (OD_580_).

### GFP fluorescence measurement

The amount of intracellular GFPmut3 was determined by fluorescence measurements. Therefore, 50 µl of each expression culture were mixed with 150 µl Tris-HCl, pH 8. Fluorescence was measured using the Tecan Infinite M1000 Pro microplate reader (Tecan, Männedorf, Swiss). The following parameters for fluorescence measurements were used: λ_Ex_ = 495 nm (bandwidth 5 nm), λ_Em_ = 505–599 nm (2 nm steps, bandwidth 5 nm, gain 100). For the calculation of relative fluorescence units, the emission maximum of GFPmut3 [24] at 511 nm was used.

### SDS-PAGE

Proteins in cell fractions and supernatants of the different expression cultures were analyzed using SDS-PAGE as described by Laemmli [34]. The extracellular proteins were precipitated using trichloroacetic acid and sodium deoxycholic acid as described in [35]. Briefly, 1 ml of culture supernatant was mixed with 100 µl of 1% sodium deoxycholic acid and incubated on ice for 10 min. 100 µl of cold 40% (v/v) trichloroacetic acid solution were added and the samples were incubated on ice for 20 min. Afterwards, the samples were centrifuged at 21,000×*g* for 30 min. The supernatant was discarded and the protein pellet was washed with 500 µl ice-cold 80% (v/v) acetone. The protein pellet was dried for 10 min and subsequently suspended in 50 mM Tris-HCl pH 8 and 2× SDS sample buffer (50 mM Tris-HCl pH 6.8, 4% (w/v) SDS, 10% (v/v) glycerol, 2% (v/v) β-mercaptoethanol, 0.03% (w/v) bromophenol blue) to an OD_580nm_ of 15. Cell fractions were diluted directly in the 2× SDS sample buffer to achieve an OD_580nm_ of 15. All samples were heated to 99 °C for 10 min. 15 µl of each sample were separated in a 16% SDS gel in a “Mini Protean II Dual Slap Cell” (BioRad, Munich, Germany) chamber for 15 min at 100 V and for 45 min at 200 V. The separated proteins were detected by staining with Coomassie Brilliant Blue [10% (w/v) ammonium sulfate, 1% phosphoric acid, 0.1% (w/v) Coomassie Brilliant Blue R-250, 20% (v/v) methanol] overnight.

### Real-time quantitative PCR for determination of transcript amount

The influence of spacer lengths on levels of transcript was analyzed for each target gene by RT-qPCR as described previously [36]. RNA was isolated from 1 ml of each expression culture using the NucleoSpin^®^ RNA Kit (Macherey-Nagel, Düren, Germany). Synthesis of cDNA was performed with 1 µg RNA with the Maxima First Strand cDNA Synthesis Kit (Thermo Fisher Scientific, St. Leon-Roth, Germany). RT-qPCR was performed using the Maxima SYBR/ROX qPCR Master Mix (Thermo Fisher Scientific, St. Leon-Roth, Germany), 50 ng cDNA of each sample and the real time qPCR primer pairs shown in Additional file 1: Table S1. Gene expression analysis was performed with the REST 2009 software (Qiagen, Hilden, Germany) using the 2^−ΔΔCT^ method with an assumed PCR efficiency of 100% [37, 38]. The expression level of the respective target gene was normalized to the level of the constitutively expressed major sigma factor gene *sigA* and compared to the expression of the same target gene with a spacer length of 4nt.

### In silico analyses

RNA stability around the translational start site was calculated as minimum free energy (MFE) by the Vienna RNAfold tool [16, 39]. The Gibbs free energy was calculated for a 39nt window which corresponds to the number of nucleotides covered by the ribosome [40]. This window was shifted between the − 50 and the + 50 nucleotide position downstream and upstream of the + 1 translational start site resulting in 62 individual MFE values for each transcript. The translation initiation rate of each mRNA was calculated using the RBS Calculator v2.0 [17, 18]. Correlation analysis of translation initiation rates with the experimentally achieved activity or fluorescence data was performed using Microsoft EXCEL 2010 (Microsoft Corporation, Redmond, Washington, USA). For calculation of the Spearman’s rank correlation coefficient (r_s_) with EXCEL, the rank of each data point was calculated using RANK.AVG function and subsequently the correlation of the ranked data was calculated using the CORREL function.

## Results and discussion

### Construction of vectors harboring spacers of different lengths

The effect of varying spacer lengths between the Shine Dalgarno (SD) sequence and start codon on the yield of protein produced by *B.* *subtilis* was analyzed with a series of vectors based on the expression vector pBSMul1. It contains the strong promoter P_*HpaII*_ [22], a strong SD sequence (TAAGGAGG), and the start codon AUG previously described as being most efficient for protein production [12]. This vector and its derivatives were successfully used in several studies for the production and secretion of recombinant proteins in *B. subtilis* [4, 5, 31, 36, 41, 42]. However, pBSMul1 contains a 4nt spacer (ACAT, Fig. 1; for convenience, pBSMul1 is named pBS4nt in this study), whereas a spacer length of 7–9nt is recommended as optimal [12]. In order to identify the optimal spacer length for expression of cytoplasmic and secreted proteins, we stepwise increased the spacer length by QuickChange^®^ PCR in pBSMul1 from 4 to 12nt by insertion of additional adenosines at the 5′ end of the spacer sequence (Fig. 1).

### Optimal spacer length increases product yield of intracellular proteins up to 27-fold depending on the target protein

To determine the effect of spacer length on the production of intracellular proteins, the genes for the intracellular model proteins GFPmut3 [24], a derivate of the green fluorescent protein from *Aequorea victoria,* and the *E. coli* β-glucuronidase UidA [25], here named GUS, were expressed from plasmids with different spacer lengths in *B. subtilis* TEB1030 [21].

**Fig. 2:**
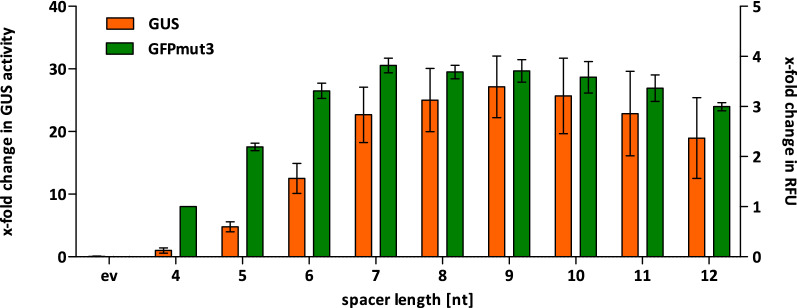
Determination of optimal spacer length for production of intracellular proteins GUS and GFPmut3. GUS activity and relative fluorescence units (RFU) for GFPmut3 were determined in cell extracts of *B.* *subtilis* TEB1030 harboring expression plasmids pBSxnt-*GFPmut3,* pBSxnt-*GUS or* the empty vector control pBSMul1 (ev) and calculated as x-fold changes compared to the basic constructs pBS4nt-*GFPmut3* or -*GUS* which was arbitrarily set to 1

We observed a moderate up to fourfold increase of GFPmut3 fluorescence and an even stronger increase of GUS activity up to 27-fold with increasing length of spacers as compared to the basic constructs with a 4nt spacer (Fig. 2). Accordingly, we also detected increasing amounts of protein by SDS-PAGE (see Additional file 1: Fig. S1A, B) indicating that longer spacers result in the production of an increased amount of protein. The optimal spacer length was at least 7nt confirming the results of a previous study using LacZ as model protein [12]. However, that study observed a peak for 7nt spacers with a decreasing productivity for longer spacers whereas our data show that product yields reach a plateau for spacers longer than 9nt. Thus, the optimal length of a spacer apparently depends on the target gene sequence as also indicated by the difference in activity increase factors determined for GFPmut3 (fourfold) and GUS (27-fold).

### Optimal spacer length depends on the N-terminal signal peptide for secreted cutinase and swollenin

Even though the ribosome binds to the 5′UTR upstream of a coding sequence, translation initiation can also be affected by the 5′end of the coding sequence itself as it is also involved in the formation of RNA secondary structures masking the ribosome binding site [43]. Genes coding for Sec- or Tat-secreted proteins contain a 5′ sequence encoding the N-terminal signal peptide [44]. Remarkably, a given signal peptide can direct the secretion of different recombinant proteins [45, 46].

**Fig. 3:**
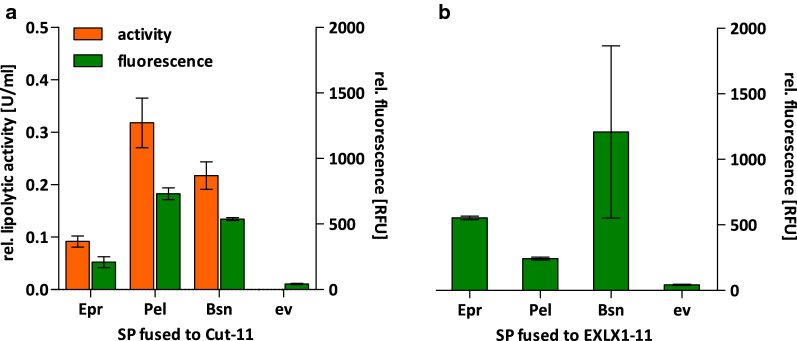
Extracellular activity and protein amount of cutinase Cut-11 and swollenin EXLX1-11 fused to different signal peptides. Relative lipolytic activity and split GFP fluorescence of Cut-11 (**a**) and EXLX1-11 (**b**) produced by *B. subtilis* TEB1030 harboring expression plasmids pBS4nt-*SPepr*-*cut*-*11/*-*EXLX1*-*11* (Epr), pBS4nt-*SPpel*-*cut*-*11/*-*EXLX1*-*11* (Pel), pBS4nt-*SPbsn*-*cut*-*11/*-*EXLX1*-*11* (Bsn), or the empty vector control pBSMul1 (ev)

To determine the effect of spacer length on the production of secreted proteins in *B.* *subtilis*, we combined three different signal peptides with two secreted model proteins, namely the homologous swollenin EXLX1 and the heterologous cutinase Cut from the fungus *F. solani pisi*. Both proteins were fused in-frame with the *B. subtilis* signal peptides of the extracellular protease Epr (SPEpr), the pectate lyase Pel (SPPel) and the extracellular ribonuclease Bsn (SPBsn). Signal peptides of Epr and Pel performed well in previous cutinase secretion screenings [4] whereas the signal peptides of Epr and Bsn improved EXLX1 secretion [31]. Both proteins were fused to a C-terminal split GFP tag (GFP11) allowing activity-independent quantification of Cut-11 and EXLX1-11 in vitro [31]. All variants were expressed in *B.* *subtilis* TEB1030 from standard plasmid pBS4nt and quantified in the culture supernatant by split GFP assay and additionally lipolytic activity assay for Cut-11 (Fig. 3). Interestingly, the secretion of Cut with the signal peptide from Epr did not result in highest cutinase activity and amount in the supernatant, although it was previously identified as the most suitable signal peptide for the secretion of cutinase [4]. This difference in secretion efficiency might be explained by the different spacer sequence used here (ACAT, see Fig. 1) and in the previous study (ATATT). Nevertheless, all three signal peptides clearly mediated secretion of both model proteins.

**Fig. 4:**
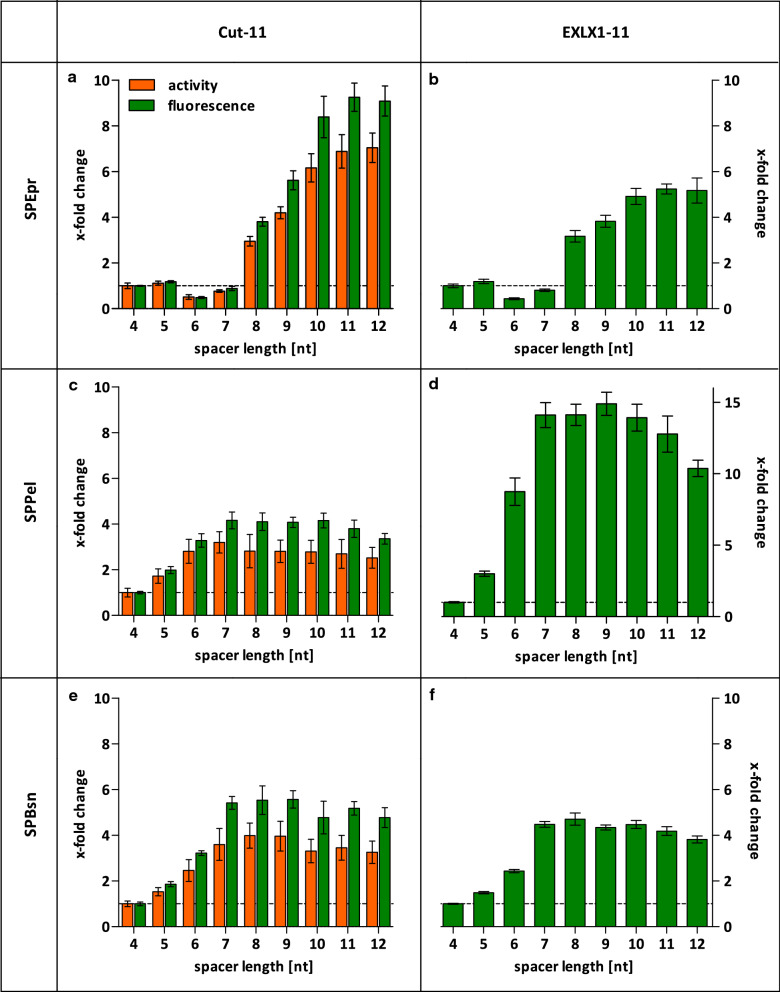
Influence of spacer length on the production of secreted proteins. X-fold change in Cut-11 activity and amount of Cut-11 and EXLX1-11 proteins determined by split GFP assay in culture supernatants of *B.* *subtilis* TEB1030 harboring expression plasmids pBSxnt-*SPepr*-*cut*-*11/*-*EXLX1*-*11* (**a**/**b**), pBSxnt-*SPpel*-*cut*-*11/*-*EXLX1*-*11* (**c**/**d**) and pBSxnt-*SPbsn*-*cut*-*11/*-*EXLX1*-11 (**e**/**f**) compared to the respective pBS4nt expression plasmid

All combinations of signal peptides and target genes were expressed with different spacer lengths in *B.* *subtilis* TEB1030 and proteins Cut-11 and EXLX1-11 were quantified in the culture supernatant by lipolytic activity and by the split GFP assay (Fig. 4). Again, all changes determined by activity and split GFP assays coincided with similar changes in protein amount detected by SDS-PAGE (Additional file 1: Fig. S1).

Interestingly, the production of Cut-11 and EXLX1-11 with the signal peptide SPEpr (Fig. 4a, b) showed a considerable decrease with spacer lengths of 6 and 7nt followed by a postponed increase of extracellular protein amount with spacer lengths of 10–12nt. This pattern was not described in previous studies dealing with spacer length optimization [12] and differs from the results obtained for both proteins fused to SPPel and SPBsn, where the optimal spacer lengths were 7–9nt with only a slight decrease for spacers with more than 10 nucleotides in length (Fig. 4c–f). Remarkably, the effects of spacer lengths on the production of the target proteins were similar for the same signal peptide indicating that the spacer length has to be adapted predominantly to the 5′ end of the gene of interest which is the SP coding sequence of Sec-secreted proteins.

As suggested in a previous study [42], we also constructed and screened a spacer library for intracellular GFPmut3 and secreted SPPel-Cut-11 with a randomized 7nt spacer NNNNCAT (N = A, T, C, G). For this, plasmids pBS7nt-*GFPmut3* and pBS7nt-*SPpel*-*cut*-*11* were mutagenized using QuikChange PCR and randomized primers introducing the NNNNCAT spacer instead of the standard AAAACAT spacer. For each protein, over 800 *B.* *subtilis* clones were cultivated and assayed in microtiter scale. The spacer sequences of 8 best performing clones were sequenced and the results were re-evaluated under shaking flask conditions with the standard constructs as reference (see Additional file 1: Fig. S2). Interestingly, protein amounts produced by the best performing clones did not exceed those obtained with the standard spacer AAAACAT.

### Spacer lengths do not directly correlate with transcript levels

The observed effects of spacer lengths on the yield of target proteins can have different reasons. On the one hand, an extension of the spacer length could influence the amount of transcript of the respective gene; on the other hand, the translation initiation rate may be affected. To distinguish between these effects, we have determined the transcript levels for different spacer lengths comprising the basic construct (4nt) and constructs with spacer lengths yielding in decreased (SPEpr 6nt) or increased (GFPmut3 7nt, GUS 10nt, SPEpr 11nt, SPPel 7nt and SPBsn 8nt) product yields (see Additional file 1: Table S2). Although seven constructs exhibited a significant (p < 0.05) change in transcript amount, the changes were only marginal (max. 3.2-fold) and do not correlate with the observed changes in product yield (see Fig. 4). For example, the transcript amount of 6nt *SPepr*-*EXLX1*-*11* was significantly increased although product yield was lower than with the basic construct. Thus, it appears that the observed changes in protein production caused by variations in spacer lengths cannot solely be explained by changed amounts of transcript. Consequently, we considered altered translation initiation efficiency as a reason for limited protein production.

### Prediction of translation initiation in silico is only partly reliable

We observed that the relation between spacer length and produced protein yielded in a similar pattern for both extracellular proteins Cut-11 and EXLX1-11 fused to the SPEpr signal peptide (Fig. 4a, b). This led us to conclude that an interaction of the spacer sequence with the 5′ region of the target gene, which encodes the signal peptide, may influence the initiation of translation. It is conceivable that mRNA secondary structures could mask the SD sequence thereby preventing ribosome binding [43]. To predict possible secondary structures masking the RBS, the minimum free energy (MFE) of a dynamic sliding 39 bp window around the translation start was calculated using the Vienna RNA Websuite [39]. This 39 bp window corresponds to the region which is occupied by the 30S subunit of the ribosome during translation initiation [40]. A high negative MFE value in this area indicates a possible secondary structure inhibiting translation initiation. The MFE values of all targets with different spacer lengths are shown in Additional file 1: Fig. S3. As the 39 bp window only embraces the signal peptide sequences of the secreted proteins (66/84/87nt for *SPpel/SPepr/SPbsn*), data for Cut-11 and EXLX1-11 were identical and are pictured only once. The mRNAs of GUS and the SPEpr variants showed very stable structures at the translation initiation site. Those target genes were also observed to need the longest spacers for optimal production yields (see Figs. 2 and 4a, b). Although the secondary structures are weakened by longer spacers in general, MFE values alone seems not to be suitable for prediction of product yields. For example, MFE-based ranking for *SPpel* predicts almost equally stable secondary structures for all spacer (Additional file 1: Fig. S3) whereas experimental data showed an increase of protein amount (Fig. 4). In addition, the atypical production pattern of SPepr-fused proteins is not reflected by the MFE values for *SPepr*.

**Fig. 5:**
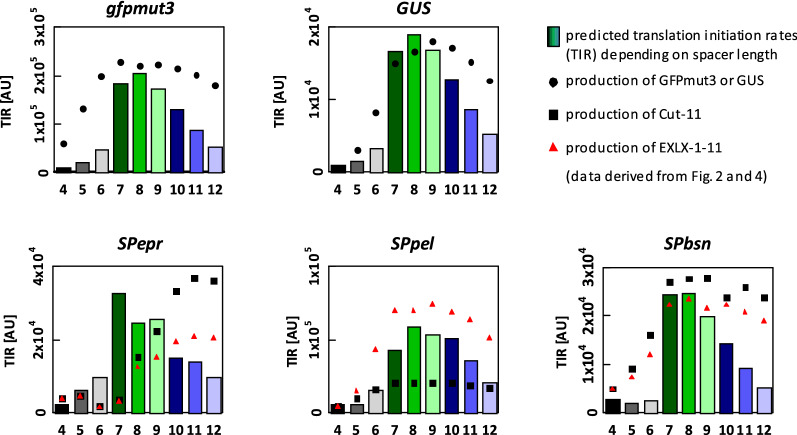
In silico analysis of translation initiation rates. Translation initiation rates (TIR, arbitrary units) for constructs with different spacer lengths were calculated using the RBS Calculator v2.0 [17, 18]. Mean values of experimentally determined changes of GFPmut3, GUS, Cut-11 and EXLX1-11 amounts (shown in Figs. 2 and 4) are indicated

A different method for the in silico analysis of translation initiation is the ‘RBS calculator’ tool [17, 18] which applies a more complex thermodynamic model to calculate the molecular interactions between mRNA and the 30S ribosomal complex for the prediction of the translation initiation rate (TIR) of a given gene [17]. We have calculated the translation initiation rates for each target with different spacer lengths (Fig. 5) using the RBS calculator V2.0 and the free energy model version 2.1. Due to the fact that the RBS calculator data is based on 35 nucleotides in front and behind the start codon, translation initiation rates for different target genes with the same signal peptide sequence are again identical. The RBS calculator data show the highest translation initiation rates for constructs with a spacer length of 7 or 8 nucleotides for all target genes (Fig. 5). Based on the RBS calculator data, the optimal spacer lengths for the production of GFPmut3, GUS and the constructs with the signal peptides SPPel and SPBsn were predictable in silico and a priori. In those cases, the variants with the predicted optimal spacer length of 8nt were among the best producing variants in our experimental setup (see also Figs. 2 and 4) showing a positive correlation with our experimental data (r_s_ between 0.70 and 0.97). In contrast, RBS calculator data did not correlate with the experimentally determined data for genes encoding the signal peptide Epr (r_s_ < 0.15).

### Analysis of a putative alternative translation start site in *SPepr*

**Fig. 6:**
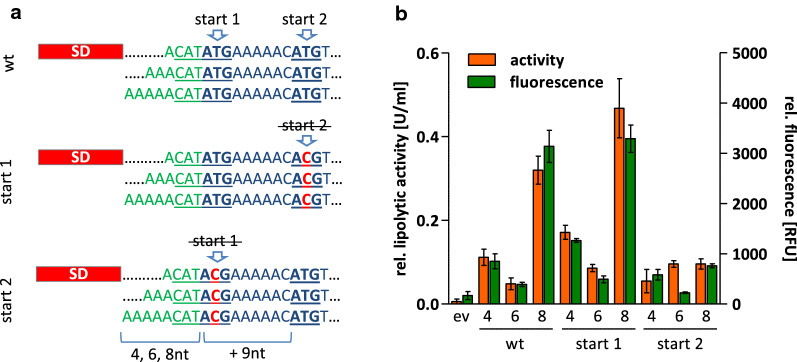
Effect of an alternative translation start site within the coding sequence of SPEpr-Cut-11. **a** The original (wt) sequence of SPEpr-Cut-11 contains an ATG codon 9nt downstream of the annotated start codon which could act as alternative translation start site and would result in a Cut-11 variant shortened by the first three amino acids of the SPEpr. Both ATG codons were individually exchanged by ACG to force translation start from the remaining ATG. **b** Lipolytic activity and amount of extracellular Cut-11 (determined by split GFP assay) which was expressed from the original plasmids (wt) with varying sizes of spacers (4, 6 or 8nt) or with first (start 2) or second ATG (start 1) codon exchanged

The atypical production patterns observed for Cut-11 and EXLX1-11 fused to the signal peptide SPEpr could neither be explained by changes in transcript level (see Additional file 1: Table S2) nor by exceptional secondary structures influencing translation initiation (Fig. 5 and Additional file 1: Fig. S3). Inspection of the coding sequence of *SPepr* identified a putative translation start site 9nt downstream of the annotated start codon (Fig. 6a). The resulting gene product would be a Cut-11 variant shortened by the first three residues of SPEpr. To analyze the effect of this alternative translation start site on SPEpr-Cut-11 production, we replaced the first and the second ATG codon, respectively, of the pBSxnt-*SPepr*-*cut*-*11* series by ACG (Fig. 6a) which is not accepted as a start codon in *B. subtilis* [47]. The resulting plasmids pBSxnt-*SPepr*-*cut*-*11*_start1 and pBSxnt-*SPepr*-*cut*-*11*_start2 were transferred into *B. subtilis* and extracellular Cut-11 production was quantified in comparison to strains harboring the original plasmids (exemplarily shown for translational start 1 with 4, 6 and 8nt, and for translational start 2 with putative 13, 15, and 17nt spacer**s** in Fig. 6b). Allowing translation to start only from the putative second start codon (start 2) resulted in low Cut-11 yields independent of the spacer length indicating that this start codon is not responsible for Cut-11 production in the “wild-type” sequence. Interestingly, forcing translation from the first start codon (start 1) still leads to an impaired Cut-11 production with a 6nt spacer but slightly increased the overall Cut-11 production by ca. 50%. Thus, the SPEpr-Cut-11 production pattern cannot be explained by the existence of a second translation start site. However, the second putative start codon together with the preceding purine-rich region (AAAAAC) may probably interact with ribosomes thereby impeding translation from the first translational start site as also discussed for additional Shine Dalgarno sequences downstream of the translational start site in *E. coli* [48].

## Conclusions

In this study, we have systematically analyzed the influence of the length of spacers located between the RBS and the start codon on the yields for intracellular (Fig. 2) and secreted proteins (Fig. 4) produced by *B. subtilis.* Apparently, varying spacer lengths had only limited influence on the transcript amount (see Additional file 1: Table S2) whereas calculated mRNA secondary structures masking the ribosome binding site (Additional file 1: Fig. S3) and translation initiation (Fig. 5) were strongly affected. The spacer sequence together with the 5′-region of the target gene which encodes the signal peptide sequence in case of Sec-secreted proteins [44], constitute the most important part of a target gene with respect to an effective translation initiation as also described recently [13]. Our results further corroborate this observation and pinpoint the importance of signal sequences not only at the level of amino acids [4] but also regarding the respective nucleotide sequences which may directly affect the efficiency of translation initiation.

Interestingly, we observed that protein yields reached a plateau when using spacers longer than at least required for optimal production, whereas literature [12] as well as in silico predictions (Fig. 5) clearly suggest a peak for 7–9nt spacers. A possible explanation is that more efficiently translated proteins could be prone to misfolding and subsequent degradation, e.g. for secreted proteins by proteases HtrA and HtrB of the general secretion stress system CssRS [49], bringing down a putative production peak for optimal spacers to the mentioned plateau level.

In summary, we have demonstrated that the length of spacer region between SD sequence and transcriptional start side plays an important role if optimal production levels of both intracellular and secreted proteins are envisaged in *B. subtilis*. In addition, the tested signal peptides seem to not only affect secretion efficiency at the protein level but also the translation initiation at mRNA level.

## Supplementary information

**Additional file 1: Fig. S1.** Influence of spacer length on the production of target proteins. **Fig. S2.** Influence of spacer composition on the production of GFPmut3 and SPPel-Cut-11. **Fig. S3.** In silico analysis of mRNA secondary structures. **Table S1.** Primers used in this study. **Table S2.** Changes in transcript amounts of target genes with different spacer lengths.

## Data Availability

All data generated or analyzed during this study are included in this article and its Additional file 1.
