# Supplementary material for: The length of ribosomal binding site spacer sequence controls the production yield for intracellular and secreted proteins by Bacillus subtilis
Source: Microb Cell Fact. 2020 Jul 29;19:154. doi: 10.1186/s12934-020-01404-2 (PMC7392706; doi:10.1186/s12934-020-01404-2)
Supplement: Supplementary file 1 — Additional file 1: Fig. S1. Influence of spacer length on the production of target proteins. Fig. S2. Influence of spacer composition on the production of GFPmut3 and SPPel-Cut-11. Fig. S3. In silico analysis of mRNA secondary structures. Table S1. Primers used in this study. Table S2. Changes in transcript amounts of target genes with different spacer lengths. [file 12934_2020_1404_MOESM1_ESM.docx]

**Additional file 1**


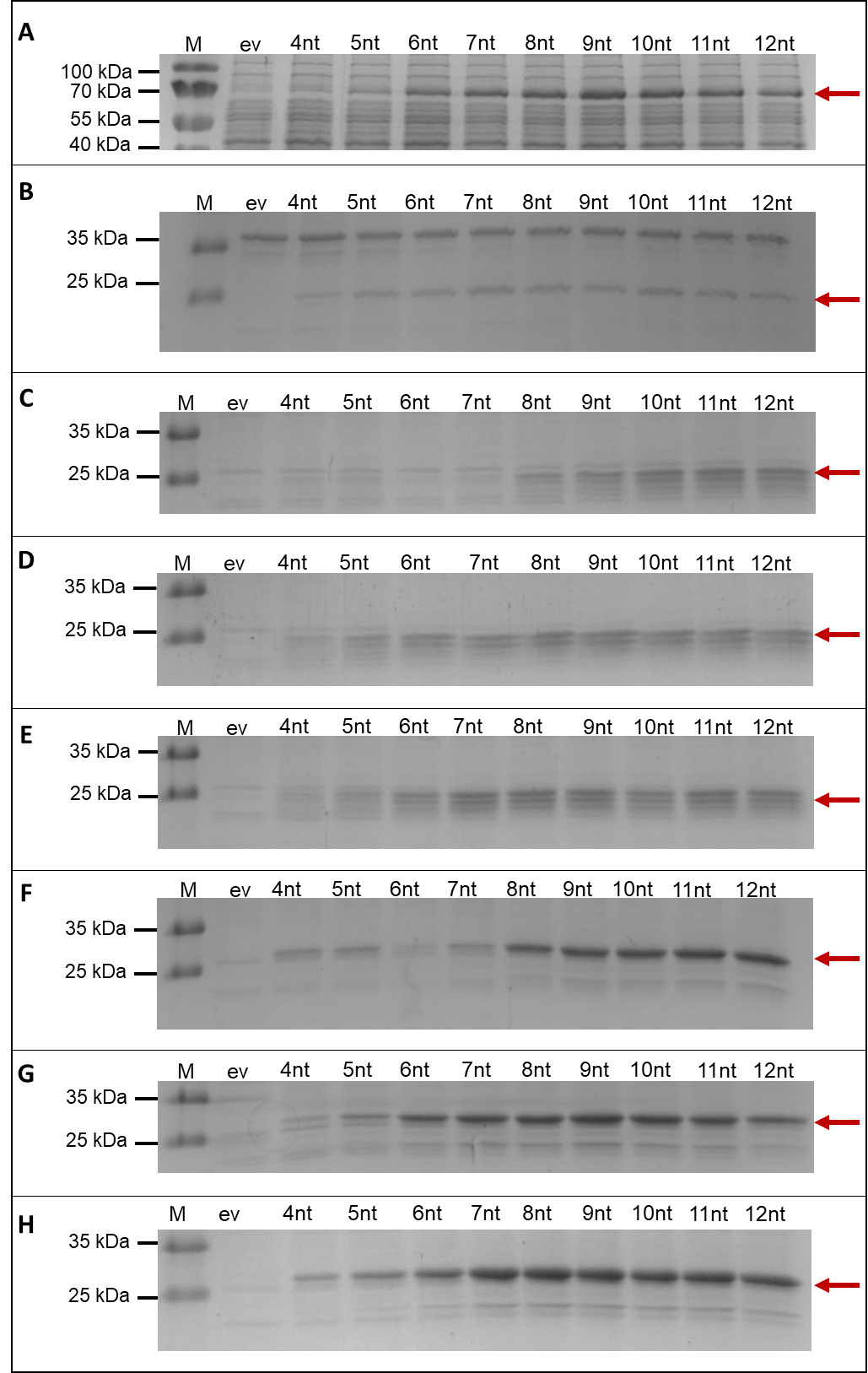


**Fig. S1: Influence of spacer length on the production of target proteins.** SDS-PAGE analysis of intracellular GUS (A) and GFPmut 3 (B) as well as extracellular SPEpr-Cut-11 (C), SPPel-Cut-11 (D), SPBsn-Cut-11 (E), SPEpr-EXLX1-11 (F), SPPel-EXLX1-11 (G), and SPBsn-EXLX1-11 (H). M: PageRuler™ Prestained Protein Ladder (Thermo Fisher Scientific). ev: empty vector control pBSMul1. The molecular weight of each target gene is indicated by the red arrow.

**Fig. S2: Influence of spacer composition on the production of GFPmut3 and SPPel-Cut-11.** X-fold change (A) in intracellular GFPmut3 fluorescence or (B) extracellular Cut-11 activity and split GFP assay of *B. subtilis* TEB1030 harboring one of the mutagenized expression plasmids with an NNNNCAT spacer compared to the optimized expression plasmid with an AAAACAT spacer*.* The spacer sequence variants were identified in a previous random mutagenesis screening.


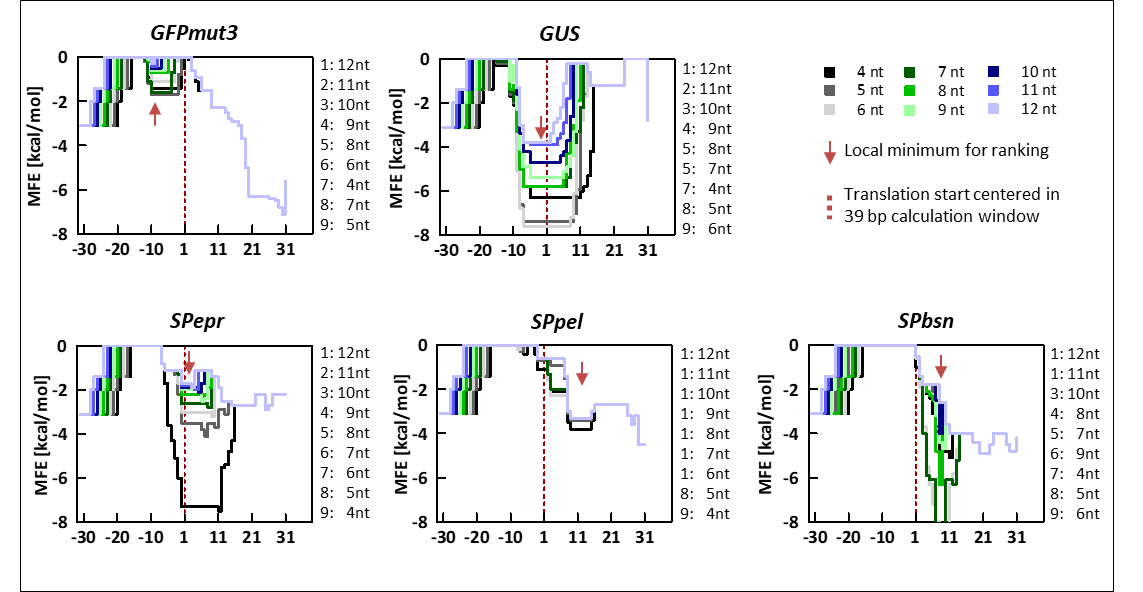


**Fig. S3: *In silico* analysis of mRNA secondary structures.** Local folding energy (minimal free energy, MFE) of a 39 bp sliding window around the start codon of the mRNA for different spacer lengths calculated for genes *gfpmut3*, *GUS* and gene fusions with signal peptide sequences *SPepr*, *SPpel*, or *SPbsn* (only the signal peptide sequence is embraced by the 39 bp window). Red arrows indicate the local minimum / most stable secondary structure that was used for ranking of spacer length.

**Table S1: Primers used in this study.**

| Name | Sequence (5` 🡪 3`, relevant sequences are underlined) | |
| --- | --- | --- |
| P1 | GAGATACCCAGATCACATGAAACAGCATGAC | Deletion of *Nde*I in pEBP41 |
| P2 | GTCATGCTGTTTCATGTGATCTGGGTATCTC |  |
| P3 | GCCCATATGCGTAAAGGAGAAGAAC | *GFPmut3* amplification |
| P4 | GGCTCTAGATTATTTGTATAGTTCATC |  |
| P5 | GCGCATATGTTACGTCCTGTAGAAAC | *GUS* (*uidA*) amplification |
| P6 | GCGCTCTAGATTATTGTTTGCCTCCCTGC |  |
| P7 | GCGCATATGAAAAACATGTCTTGCAAAC | 5´-*SPepr* |
| P8 | GCGCATATGAAAAAAGTGATGTTAGCTAC | 5´-*SPpel* |
| P9 | GCGCATATGACAAAAAAAGCATGGTTTC | 5´-*SPbsn* |
| P10 | TTTCGACCTCTAGATTATGTGATGCC | 3´-*GFP11* tag |
| P11 | GATTTACATAATAAGGAGGAACATATGAAAAACATG | 5nt spacer |
| P12 | CATGTTTTTCATATGTTCCTCCTTATTATGTAAATC |  |
| P13 | GATTTACATAATAAGGAGGAAACATATGAAAAACATG | 6nt spacer |
| P14 | CATGTTTTTCATATGTTTCCTCCTTATTATGTAAATC |  |
| P15 | GATTTACATAATAAGGAGGAAAACATATGAAAAACATG | 7nt spacer |
| P16 | CATGTTTTTCATATGTTTTCCTCCTTATTATGTAAATC |  |
| P17 | GATTTACATAATAAGGAGGAAAAACATATGAAAAACATG | 8nt spacer |
| P18 | CATGTTTTTCATATGTTTTTCCTCCTTATTATGTAAATC |  |
| P19 | GATTTACATAATAAGGAGGAAAAAACATATGAAAAACATG | 9nt spacer |
| P20 | CATGTTTTTCATATGTTTTTTCCTCCTTATTATGTAAATC |  |
| P21 | GATTTACATAATAAGGAGGAAAAAAACATATGAAAAACATG | 10nt spacer |
| P22 | CATGTTTTTCATATGTTTTTTTCCTCCTTATTATGTAAATC |  |
| P23 | GATTTACATAATAAGGAGGAAAAAAAACATATGAAAAACATG | 11nt spacer |
| P24 | CATGTTTTTCATATGTTTTTTTTCCTCCTTATTATGTAAATC |  |
| P25 | GATTTACATAATAAGGAGGAAAAAAAAACATATGAAAAACATG | 12nt spacer |
| P26 | CATGTTTTTCATATGTTTTTTTTTCCTCCTTATTATGTAAATC |  |
| P27 | GATTTACATAATAAGGAGGNNNNCATATGCGTAAAGGAG | random spacer for *GFPmut3* |
| P28 | CTCCTTTACGCATATGNNNNCCTCCTTATTATGTAAATC |  |
| P29 | GATTTACATAATAAGGAGGNNNNCATATGAAAAAAGTG | random spacer for *SPpel-cut-11* |
| P30 | CACTTTTTTCATATGNNNNCCTCCTTATTATGTAAATC |  |
| P31 | AATAAGGAGGACATACGAAAAACATGTCTTG | exchange of first ATG in pBS4nt-*SPepr-cut-11* |
| P32 | CAAGACATGTTTTTCGTATGTCCTCCTTATT |  |
| P33 | ATAAGGAGGAACATACGAAAAACATGTCTTG | exchange of first ATG in pBS5nt-*SPepr-cut-11* |
| P34 | CAAGACATGTTTTTCGTATGTTCCTCCTTAT |  |
| P35 | TAAGGAGGAAACATACGAAAAACATGTCTTG | exchange of first ATG in pBS6nt-*SPepr-cut-11* |
| P36 | CAAGACATGTTTTTCGTATGTTTCCTCCTTA |  |
| P37 | AAGGAGGAAAACATACGAAAAACATGTCTTG | exchange of first ATG in pBS7nt-*SPepr-cut-11* |
| P38 | CAAGACATGTTTTTCGTATGTTTTCCTCCTT |  |
| P39 | AGGAGGAAAAACATACGAAAAACATGTCTTG | exchange of first ATG in pBS8nt-*SPepr-cut-11* |
| P40 | CAAGACATGTTTTTCGTATGTTTTTCCTCCT |  |
| P41 | GGAGGAAAAAACATACGAAAAACATGTCTTG | exchange of first ATG in pBS9nt-*SPepr-cut-11* |
| P42 | CAAGACATGTTTTTCGTATGTTTTTTCCTCC |  |
| P43 | GAGGAAAAAAACATACGAAAAACATGTCTTG | exchange of first ATG in pBS10nt-*SPepr-cut-11* |
| P44 | CAAGACATGTTTTTCGTATGTTTTTTTCCTC |  |
| P45 | AGGAAAAAAAACATACGAAAAACATGTCTTG | exchange of first ATG in pBS11nt-*SPepr-cut-11* |
| P46 | CAAGACATGTTTTTCGTATGTTTTTTTTCCT |  |
| P47 | GGAAAAAAAAACATACGAAAAACATGTCTTG | exchange of first ATG in pBS12nt-*SPepr-cut-11* |
| P48 | CAAGACATGTTTTTCGTATGTTTTTTTTTCC |  |
| P49 | ACATATGAAAAACACGTCTTGCAAACTTGT | exchange of second ATG in pBSxnt-*SPepr-cut-11* |
| P50 | ACAAGTTTGCAAGACGTGTTTTTCATATGT |  |
| Real time qPCR primers | | |
| RT-GFPmut3-fwd | GCGATGGCCCTGTCCTTTTACC | |
| RT-GFPmut3-rev | TGCCATGTGTAATCCCAGCAGC | |
| RT-Gus-fwd | TCTGCGACGCTCACACCGAT | |
| RT-Gus-rev | GCCGTTTCCAAATCGCCGCT | |
| RT-Cut-fwd | AGAACCTACAGAACCGTGGCCG | |
| RT-Cut-rev | AAGCCAAGTGAGGTGCAGCAA | |
| RT- EXLX1-fwd | ACTGACATCCGCGGAAAAGTTGT | |
| RT- EXLX1-rev | AGCCGCCATCAGAGCCGATA | |
| RT-SigA-fwd | ATCGCCTGTCTGATCCACCA | |
| RT-SigA-rev | GGTATGTCGGACGCGGTATG | |

**Table S2 Changes in transcript amounts of target genes with different spacer lengths.** Relative changes with lower and upper range of transcript level of *GUS, GFPmut3*, *cut-11* and *EXLX1-11* (the latter two with different signal peptide sequences) in strains harboring the standard expression plasmids (4nt) and plasmids with different spacer lengths (see text for further information). Significantly increased transcript levels (p < 0.05) are marked with asterisks.

| target gene | spacer length [nt] | rel. change | lower range | upper range |
| --- | --- | --- | --- | --- |
| *GUS* | 4 | 1 | 0.56 | 1.80 |
|  | 10 | 2.23* | 1.36 | 3.64 |
| *GFPmut3* | 4 | 1 | 0.31 | 3.20 |
|  | 7 | 2.22* | 0.89 | 5,54 |
| *SPepr-cut-11* | 4 | 1 | 0.36 | 2.76 |
|  | 6 | 1.09 | 0.45 | 2.64 |
|  | 11 | 2.06* | 0.96 | 4.41 |
| *SPpel-cut-11* | 4 | 1 | 0.52 | 1.91 |
|  | 7 | 2.01 | 0.75 | 5,41 |
| *SPbsn-cut-11* | 4 | 1 | 0.72 | 1.39 |
|  | 8 | 1.63 | 0.78 | 3.39 |
| *SPepr-EXLX1-11* | 4 | 1 | 0.49 | 2.05 |
|  | 6 | 1.60* | 0.89 | 2,89 |
|  | 11 | 3.23* | 1.42 | 7.47 |
| *SPpel-EXLX1-11* | 4 | 1 | 0.73 | 1.37 |
|  | 7 | 1.58* | 0.95 | 2.64 |
| *SPbsn-EXLX1-11* | 4 | 1 | 0.59 | 1.71 |
|  | 8 | 1.39* | 0.94 | 2.06 |
